# A highly sensitive fluorimetric method for determination of lenalidomide in its bulk form and capsules via derivatization with fluorescamine

**DOI:** 10.1186/1752-153X-6-118

**Published:** 2012-10-16

**Authors:** Ibrahim A Darwish, Nasr Y Khalil, Ahmed H Bakheit, Nourh Z Alzoman

**Affiliations:** 1 Department of Pharmaceutical Chemistry, College of Pharmacy, King Saud University,, P.O. Box 2457, Riyadh, 11451, Saudi Arabia

**Keywords:** Lenalidomide, Fluorescamine, Flourimetry, Pharmaceutical analysis

## Abstract

**Background:**

Lenalidomide (LND) is a potent novel thalidomide analog which demonstrated remarkable clinical activity in treatment of multiple myeloma disease via a multiple-pathways mechanism. The strong evidences-based clinical success of LND in patients has led to its recent approval by US-FDA under the trade name of Revlimid® capsules by Celgene Corporation. Fluorimetry is a convenient technique for pharmaceutical quality control, however there was a fluorimetric method for determination of LND in its bulk and capsules.

**Results:**

A novel highly sensitive and simple fluorimetric method has been developed and validated for the determination of lenalidmide (LND) in its bulk and dosage forms (capsules). The method was based on nucleophilic substitution reaction of LND with fluorescamine (FLC) in aqueous medium to form a highly fluorescent derivative that was measured at 494 nm after excitation at 381 nm. The factors affecting the reaction were carefully studied and optimized. The kinetics of the reaction was investigated, and the reaction mechanism was postulated. Under the optimized conditions, linear relationship with good correlation coefficient (0.9999) was found between the fluorescence intensity and LND concentration in the range of 25–300 ng/mL. The limits of detection and quantitation for the method were 2.9 and 8.7 ng/mL, respectively. The precision of the method was satisfactory; the values of relative standard deviations did not exceed 1.4%. The proposed method was successfully applied to the determination of LND in its bulk form and pharmaceutical capsules with good accuracy; the recovery values were 97.8–101.4 ± 1.08–2.75%.

**Conclusions:**

The proposed method is selective and involved simple procedures. In conclusion, the method is practical and valuable for routine application in quality control laboratories for determination of LND.

## Background

Lenalidomide (LND) is a potent novel thalidomide analog which demonstrated remarkable clinical activity in treatment of multiple myeloma disease [1-5] via a multiple-pathways mechanism [6-9]. The strong evidences-based clinical success of LND in patients has led to its recent approval by US-FDA under the trade name of Revlimid® capsules by Celgene Corporation [10]. LND has an improved side effects profile than its parent compound thalidomide [11]. These side effects can be managed by combination therapy and/or careful dose adjustment [12]. The therapeutic benefits profile of LND is anticipated to encourage the development of new pharmaceutical preparations for LND. As a consequence, there is an increasing demand for proper analytical technologies for quality assurance of LND formulations.

Few methods have been reported for the determination LND in bulk material and in capsules. These methods included two spectrophotometric methods [13]. The first method was based on diazo-coupling reaction with N-(1-napthyl) ethylenediamine dihydrochloride and the second method was based on the formation of a colored condensation product with p-dimethylaminocinnamaldehyde. In addition, two HPLC methods have reported for analysis of bulk material of LND and its related impurities [14] and capsules [15]. These methods were associated with some major drawbacks such as lack of selectivity, time-consumption and/or use of expensive instruments.

Fluorimetry is considered one of the most convenient analytical techniques, because of its inherent simplicity, high sensitivity, low cost, and wide availability in most quality control laboratories. No attempt has yet been made for the fluorimetric determination of LND. The present study describes, for the first time, the development of a novel highly sensitive and simple fluorimetric method for the determination of LND in its bulk form and capsules. The method was based on the derivatization of LND with fluorescamine (FLC) in aqueous medium to produce a highly fluorescent product that was measured fluorimetrically at 494 nm after excitation at 381 nm.

## Experimental

### Apparatus

Fluorescence measurements were carried out on a RF-5301 PC spectrofluorimeter (Shimadzu Corporation Kyoto, Japan) equipped with a 150 W xenon lamp and 1 cm quartz cells. The slit widths of both the excitation and emission monochromators were set at 1.5 nm. The calibration and linearity of the instrument were frequently checked with standard quinine sulphate. pH meter Model 211 a product of HANNA Instruments Inc. (Smithfield, RI, USA).

### Reagents and materials

Lenalidomide (LND), free base (3-(4`-aminoisoindoline-1`-one)-1-piperidine-2,6-dione) (LC Laboratories®, Woburn, MA, USA ) was obtained and used as received; its purity was 100.2 ± 1.25%. Fluorescamine (FLC; Sigma Chemical Co., St. Louis, USA) was prepared in acetonitrile to contain 0.025% (w/v); the solution could be used for seven days when kept in the refrigerator. Revlimid® capsules (Celgene Corporation, New Jersy, USA) labeled to contain 5 mg LND per capsule was obtained from the local market. Double distilled water was obtained through WSC-85 water purification system (Hamilton Laboratory Glass Ltd., Kent, USA), and used throughout the work. All other solvents and materials used throughout this study were of analytical grade.

### Preparation of standard and sample solutions

#### Lenalidomide standard solution

An accurately weighed amount (25 mg) of LND was quantitatively transferred into a 25-mL calibrated flask, dissolved in 20 mL methanol, completed to volume with the same solvent to obtain a stock solution of 1 mg/mL. This stock solution was further diluted with water to obtain a working stock solution containing 0.5 μg/mL.

#### Capsules sample solution

The contents of 20 Revlimid® capsules (Celgene Corporation, NJ, USA), labeled to contain 5 mg of LEN per capsule were evacuated and weighed. An accurately weighed portion equivalent to 50 mg of LND was transferred into a 50-mL calibrated flask containing ~ 40 mL of methanol. The contents of the flask were swirled, sonicated for 5 min, and then completed to volume with methanol. The contents were mixed well and filtered rejecting the first portion of the filtrate. The prepared solution was diluted quantitatively with distilled water to obtain a suitable concentration for the analysis.

### General recommended procedure

Accurately measured aliquots of LND working stock solution (0.5 μg/mL) were transferred into separate 10-mL calibrated flasks to obtain a series of LND standard solutions covering the working range of 25–300 ng/mL in the final solution. One milliliter of FLC solution (0.025% w/v) was added to each flask. The reaction was allowed to proceed at room temperature (25 ± 2°C) for 10 min, and then completed to volume with water. The fluorescence intensity of the resulting solutions were measured at 494 nm after excitation at 381 nm against a reagent blank prepared in the same manner but using water instead of the LND working stock solution.

### Determination of stoichiometric ratio

The Job’s method of continuous variation [16] was employed. Master equimolar (1.5×10^−5^ M) aqueous solutions of LND and FLC were prepared. Series of 10-mL portions of the master solutions of LND and FLC were made up comprising different complementary proportions (0:10, 1:9, . . ., 9:1, 10:0, inclusive) in 10-mL calibrated flasks and the reactions were allowed to proceed for 10 min. The solutions were further manipulated and the fluorescence signals were measured as described under the general recommended procedure.

## Results and discussion

### Excitation and emission spectra

**Figure 1:**
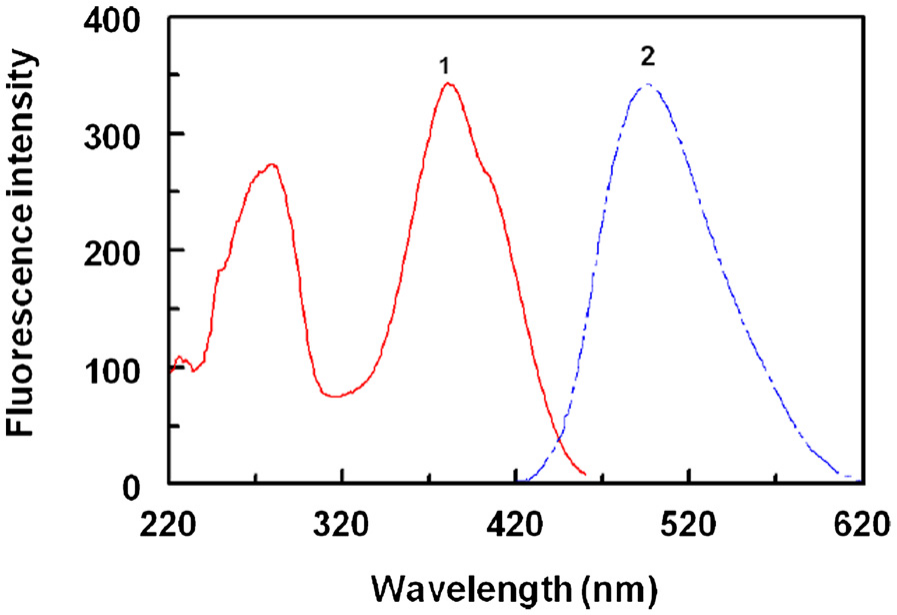
Excitation (1) and emission (2) spectra of the reaction product of LND (275 ng/mL) with FLC (0.025%, w/v).

Because of the absence of native fluorescence of LND, its derivatization with fluorogenic reagent was necessary for its fluorimetric determination. FLC was chosen as a derivatizing reagent because it forms highly fluorescent derivatives with primary amines under relatively mild reaction conditions [17]. It was found that LND reacts with FLC and forms a highly fluorescent derivative that exhibited maximum fluorescence intensity (λem) at 494 nm after excitation at wavelength (λex) of 381 nm. The excitation and emission spectra for the reaction product of LND with FLC are given in Figure 1.

### Optimization of reaction variables

#### Effect of FLC concentration

**Figure 2:**
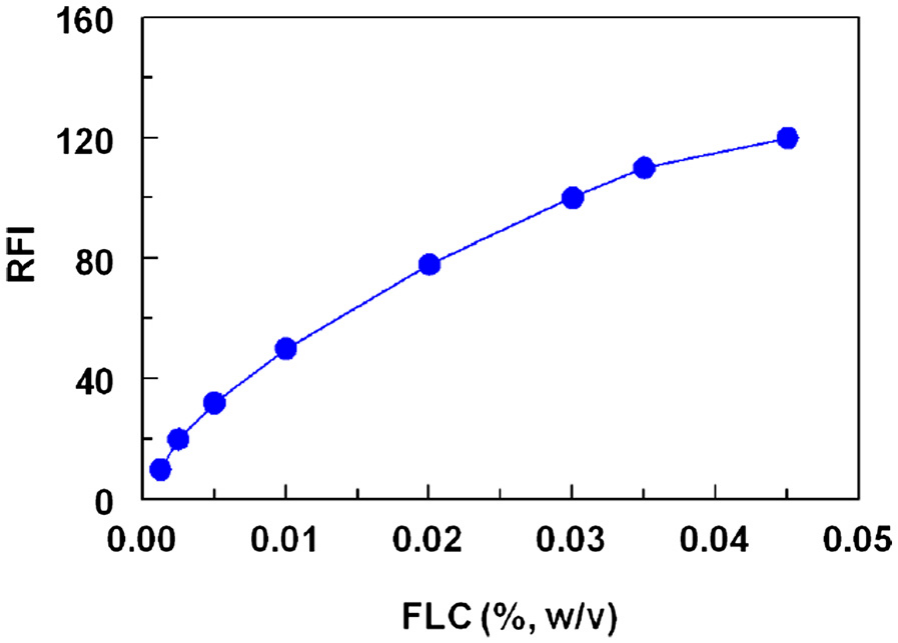
Effect of FLC concentration on its reaction with LND (75 ng/mL).

The study of the reaction between LND and FLC revealed that the reaction was dependent on the FLC concentration as the relative fluorescence intensity (RFI) of the reaction mixture increased steadily as the FLC concentration increased up to a final concentration of 0.002%, w/v (Figure 2). Beyond this concentration, the slope of the curve significantly decreased. For more precise readings, a concentration of 0.025% (w/v) of FLC reagent solution was used throughout the further experiments.

#### Effect of pH

**Figure 3:**
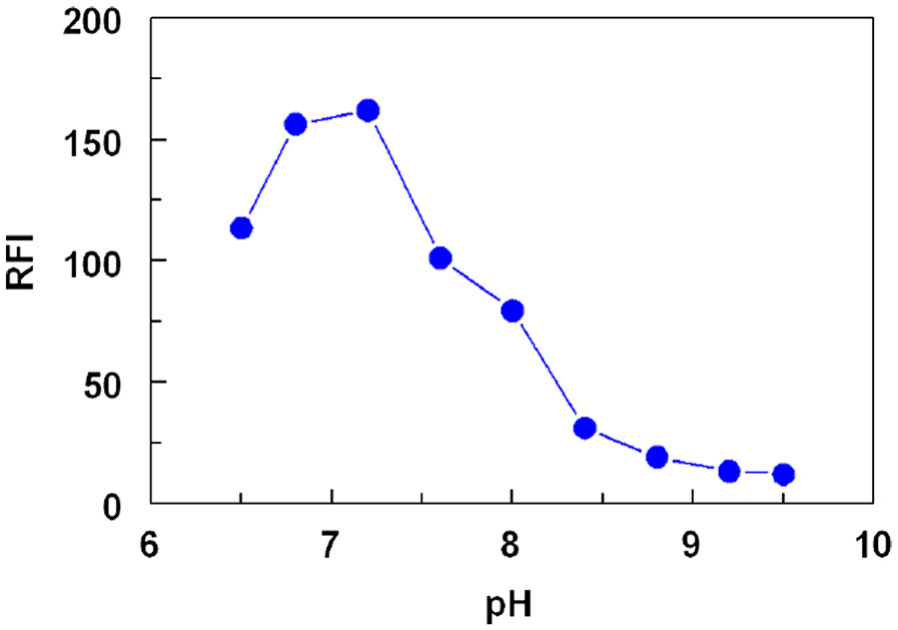
Effect of pH on the reaction of FLC (0.025%, w/v) with LND (130 ng/mL).

The effect of the pH on the reaction was studied by carrying out the reaction in borate buffer solution in the pH range of 6.5–9.5. The results indicated that the RFI increased initially as the pH increased and maximum readings were attained at pH 7.0 ± 0.2 (Figure 3). In previous studies involving FLC as a fluorofore, the maximum readings were obtained at pH around 8.0 [18]. This was possibly due to the predominance of the free amino group of the investigated substance rather than its salt form in acidic pH. Consequently, this facilitates the nucleophilic substitution reaction. In the present study, such alkaline pH was not necessary because the LND is already in the form of free base. Furthermore, at higher pH values, sharp decrease in the readings occurred (Figure 3). This was probably attributed to the hydrolysis of the reaction product between LND and FLC in alkaline medium. Neutral pH was found to be optimum for the reaction between LND and FLC. Distilled water was compared with borate buffer of pH 7, and similar results were obtained. Therefore, the reactions in all the subsequent experiments were carried out in distilled water. This was in favor of the simplicity of the proposed procedure, and environmental and health safety.

#### Effect of time

**Figure 4:**
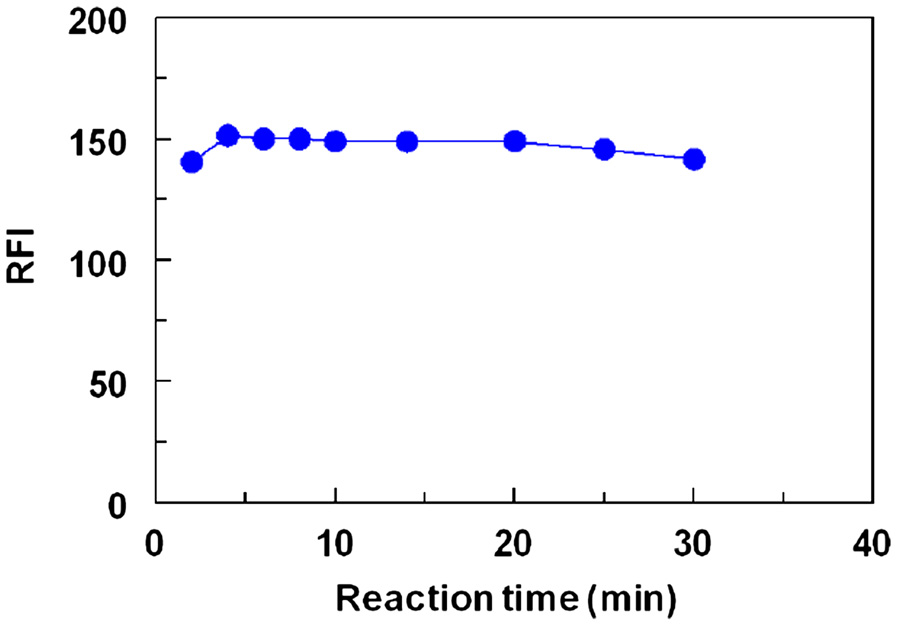
Effect of time on the reaction of FLC (0.025%, w/v) with LND (130 ng/mL).

In order to determine the optimum time required for completion of the reaction, the derivatization reaction was carried out at room temperature (25 ± 2°C) and the induced fluorescence signals were measured immediately after the addition of FLC and monitored for 30 min. The optimum reaction time was considered as the time at which the highest fluorescence signals with reproducible results are obtained in a comfortable measurement region on the FI-time curve (wide plateau). The results indicated that the reaction was very fast and almost completed within 5 min (Figure 4). Beyond this time, the RFI values did not change by time. For comfortable readings with high precise results, all the subsequent reactions were carried out for 10 min.

#### Effect of diluting solvent

**Figure 5:**
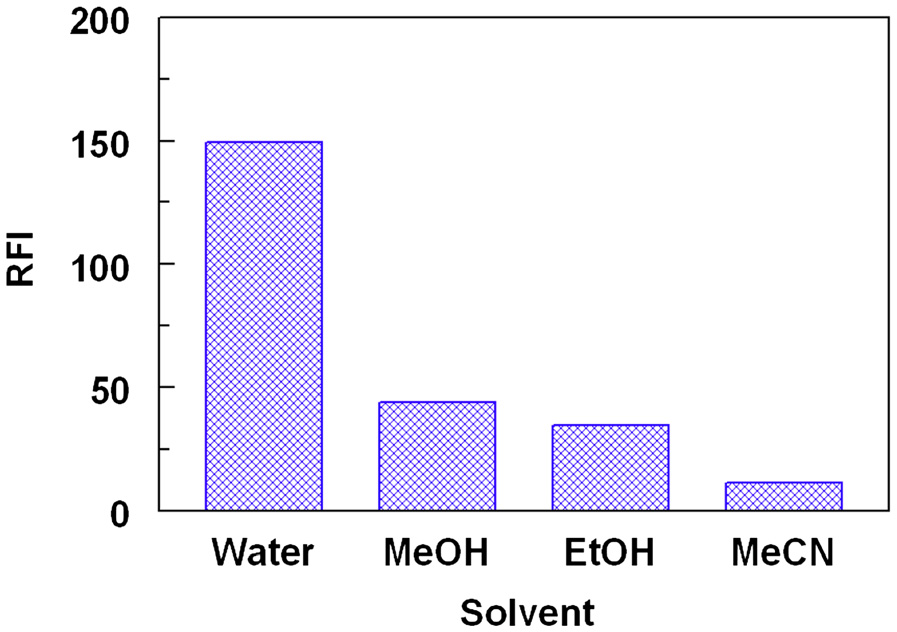
**Effect of diluting solvent on the reaction between LND (130 ng/mL) and FLC (0.025%, w/v).** Solvents were: water, methanol (MeOH), ethanol (EtOH), and acetonitrile (MeCN).

In order to select the most suitable diluting solvents for the formation and stability of the reaction product, different solvent were investigated. These solvents were: water, methanol, ethanol, and acetonitrile. The highest fluorescence intensities were obtained when water was used as a diluting solvent (Figure 5).

#### Stability of the fluorescent derivative

The effect of time on the stability of the LND-FLC fluorescent derivative was studied by monitoring the fluorescence intensities of the reaction solution (after dilution) at different time intervals. It was found that the RFI values remain constant for at least 1 hour. This allowed the processing of large batches of samples, and their comfortable measurements with convenience. This increased the convenience of the method as well as made it applicable for large number of samples in quality control laboratories.

A summary for the optimization of the variables affecting the reaction of LND with FLC is given in Table 1.

**Table 1:** Optimization of variables affecting the reaction of LND with FLC

**Variable**	**Studied range**	**Optimum condition**
FLC conc. (%, w/v)	0.00025 – 0.0045	0.0025
pH	6.5–9.5	7 ± 0.2
Reaction time (min)	2–30	10
Solvent	Different ^a^	Water
Stability of LND-FLC (min)	10–60	10–60
Excitation wavelength, λ_ex_ (nm)	220–460	381
Emission wavelength, λ_em_ (nm)	420–620	494

^a^ Solvents tested: Water, methanol, ethanol, and acetonitrile.

^b^ The stability of the LND-FLC was studied after dilution of the reaction solution.

### Stoichiometry, kinetics and mechanism of the reaction

**Figure 6:**
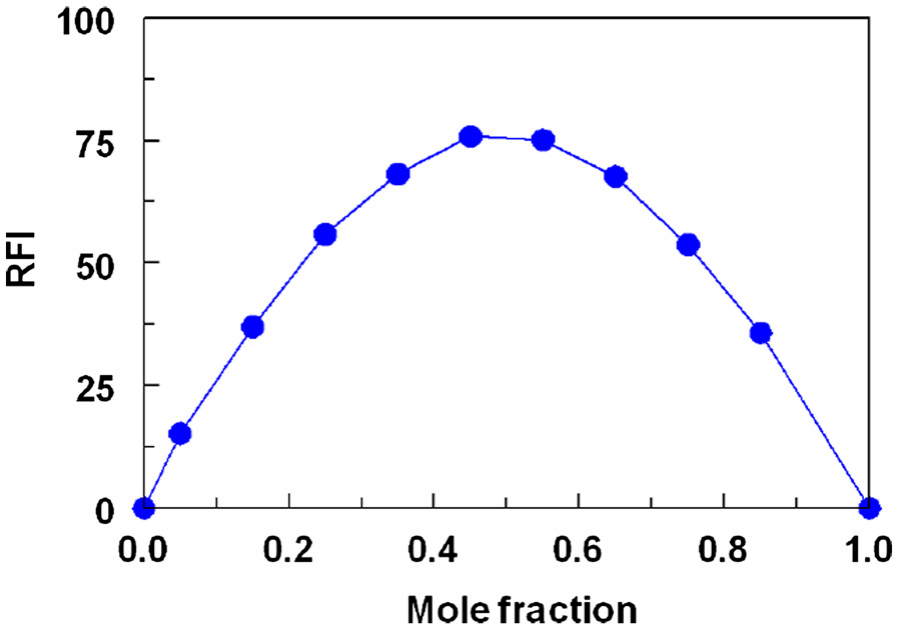
Job’s plot for the reaction between LND and FLC.

**Figure 7:**
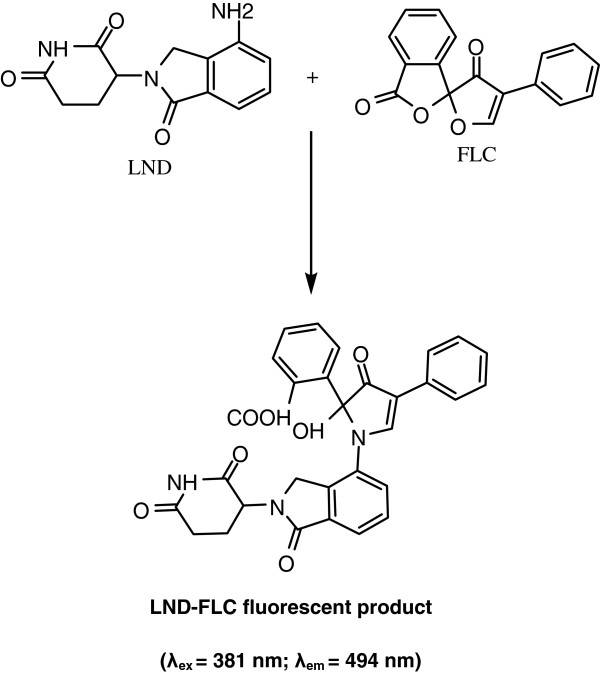
Scheme for the reaction pathway between LND and FLC.

Under the optimum conditions (Table 1), the stoichiometry of the reaction between LND and FLC was investigated by Job’s method [16]. The symmetrical bell-shape of Job’s plot (Figure 6) indicated that the LND:FLC ratio was 1:1. Based on this ratio, the reaction pathway was postulated to be proceeded as shown in Figure 7.

**Figure 8:**
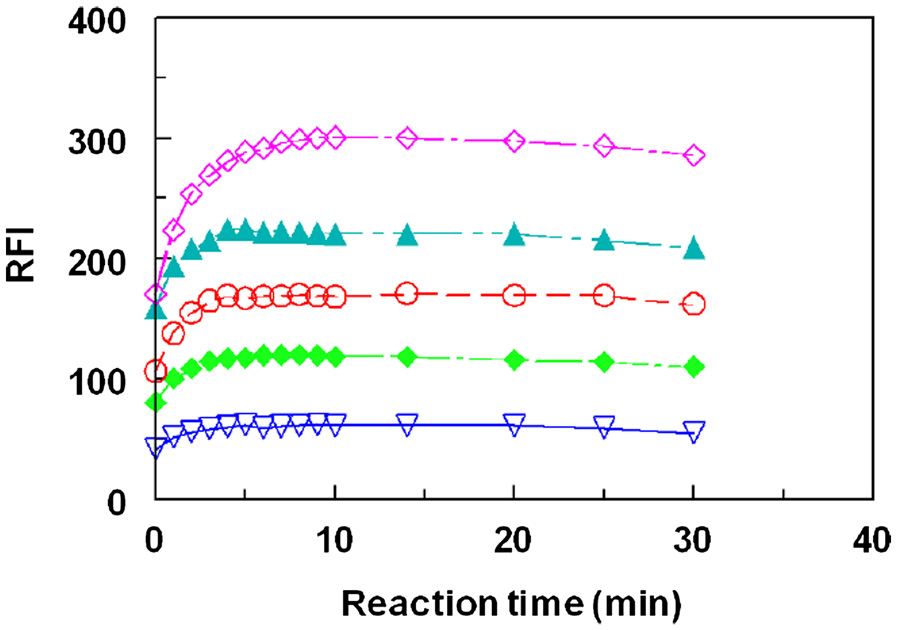
**Relative fluorescence intensity (RFI)-time curves for the reaction of a fixed concentration of FLC with varying concentrations of LND.** LND concentrations were 45 (▽), 95 (♦), 130 (○), 185 (▲), and 260 (□) ng/mL.

**Figure 9:**
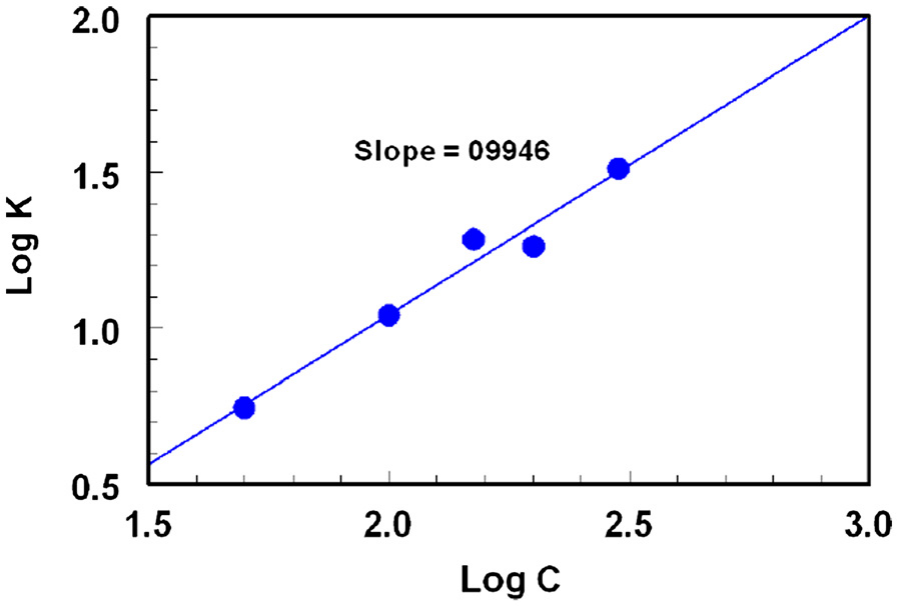
Linear plot for Log C versus Log K for the kinetic reaction of LND and FLC.

Under the optimum conditions (Table 1), the RFI-time curves for the reaction at varying LND concentrations (1.93×10^–7^ – 1.16×10^–6^ M)] with a fixed concentration of FLC [9×10^–5^ M)] were generated (Figure 8). The initial reaction rates (K) were determined from the slopes of these curves. The logarithms of the reaction rates (Log K) were plotted as a function of logarithms of LND concentrations (log C); Figure 9. The regression analysis for the values was performed by fitting the data to the following equation:

(1) Log K=log K′+nlogC

where K is reaction rate, *K*′ is the rate constant, *C* is the molar concentration of LND, and *n* (slope of the regression line) is the order of the reaction. As seen in Figure 9, a straight line with slope values of 0.9946 (≈ 1) confirming that the reaction was first order. However under the optimized reaction conditions, the concentration of FLC was in much more excess than that of LND in the reaction solution. Therefore, the reaction was regarded as a pseudo-first order reaction.

### Validation of the method

#### Calibration and sensitivity

Under the optimum conditions (Table 1), calibration curve for the determination of LND by its reaction with FLC was constructed by plotting the RFI as a function of the corresponding LND concentration. The regression equation for the results was: RFI = a + bC, where RFI is the relative fluorescence intensity, C is the concentration of LND in ng/mL. Linear relationship with small intercept and excellent correlation coefficient (r = 0.9999) was obtained in the range of 25–300 ng/mL. The limit of detection (LOD) and limit of quantification (LOQ) were determined according to ICH guidelines for validation of analytical procedures [19]. The LOD and LOQ values were found to be 2.9 and 8.7 ng/mL, respectively. The parameters for the analytical performance of the proposed fluorimetric method are summarized in Table 2.

**Table 2:** Statistical parameters for the determination of LND by the proposed fluorimetric method based on its reaction with FLC

**Parameter**	**Value**
Linear range (ng/mL)	25–300
Intercept	11.8
SD of intercept	0.921
Slope	1.047
SD of slope	0.0054
Correlation coefficient (r)	0.9999
LOD (ng/mL)	2.9
LOQ (ng/mL)	8.7

#### Accuracy and precision

The accuracy and precision of the proposed fluorimetric method was determined by replicate analysis of five different concentrations of the working standard. The recovery values were 97.8-101.4 ± 1.08 - 2.75% (Table 3), indicating the accuracy of the proposed method. The average recovery from all the concentrations was found to be 99.5% with SD of 1.40% indicating the good accuracy and reproducibility of the results. Furthermore intra- and inter-day precisions for determination of LND in bulk powder were assessed at three varying LND concentrations (low, medium, and high). The average recovery values were 101.40 and 102.27% with RSD values of 1.13 and 2.29% for intra- and inter-assay precision, respectively (Table 4). These good recovery values and low RSD values revealed the high accuracy and precisions, respectively.

**Table 3:** Recovery studies for determination of LND by the proposed fluorimetric method based on its reaction with FLC

**Taken (ng/mL)**	**Recovery (% ± SD)**^**a**^
100	98.5 ± 2.54
150	97.8 ± 2.02
200	100.1 ± 1.24
250	99.5 ± 2.75
300	101.4 ± 1.08
	Average 99.46 ± 1.40

^**a**^ Values are mean of three determinations.

**Table 4:** Intra-assay and inter-assay precision and accuracy for determination of LND by the proposed fluorimetric method

**Nominal conc. (ng/mL)**	**Intra-assay**		**Inter-assay**	
	**Measured conc. (ng/mL)**	**Recovery (% ± RSD)**^**a**^	**Measured conc. (ng/mL)**	**Recovery (% ± RSD)**^**b**^
25	25.60	102.40 ± 2.86	26.10	104.44 ± 0.69
100	100.56	100.56 ± 0.67	102.50	102.50 ± 2.23
300	310.50	100.35 ± 0.68	299.6	99.87 ± 0.39
	Average	101.10 ± 1.13	Average	102.27 ± 2.29

^**a**^ Values are mean of five determinations.

^**b**^ Values are mean of four determinations.

#### Robustness and ruggedness

Robustness was examined by evaluating the influence of small variation in the method variables on its analytical performance. In these experiments, one parameter was changed whereas the others were kept unchanged, and the recovery percentage was calculated each time. It was found that variation in the FLC concentrations (0.02–0.03%, w/v), temperature (optimum ± 2°C), pH (6.8 - 7.2) and time (optimum ± 5 min) did not significantly affect the recovery values. The most critical factor affecting the results was the FLC concentration and therefore it had to be added accurately to attain a fixed concentration in all the solutions. Ruggedness was also tested by applying the method to the assay of LND using fixed operational conditions within ± 10% changes and on different days. The results were reproducible and the RSD% did not exceed 2.29%.

### Practical applications of the proposed method

It is evident from the above-mentioned results that the proposed method gave satisfactory results with LND in bulk form. Also the pharmaceutical dosage forms (Revlimid® capsules) were analyzed for their LND content by the proposed method. The percentage found from the label claim was 100.11 ± 1.61% of the label claim, indicating the successful applicability of the proposed method in quality control laboratories for determination of LND. The results obtained by the proposed method was compared with those obtained by a reported method [15] with respect to the accuracy (by t-test), and precision (by F-test). It was found that the calculated t- and F-values were lower than the tabulated ones. This indicated that there were no significant differences between the means and variance between the two methods in terms of the accuracy and precision.

### Advantages of the proposed method over the reported methods

This study represents the first report describing the successful evaluation of FLC as an analytical reagent in the development of a highly sensitive and simple fluorimetric method for the quantitative determination of LND. The proposed method is superior to the previously reported spectrophotometric methods in terms of the sensitivity and simplicity of the derivatization procedures. As well, the proposed procedure used water as a green, inexpensive, and safe solvent, rather than the costive and toxic organic solvents that have been employed in the previously reported HPLC methods. In addition, the method employed a simple inexpensive fluorimeter that is available in most quality control laboratories, rather than the expensive HPLC systems.

## Conclusions

A novel simple and sensitive fluorimetric method for the determination of LND in bulk form and capsules has been successfully developed and validated. The method involved simple derivatization of LND with FLC reagent, and subsequent measurement of the fluorescence intensity of the fluorescent reaction product. The proposed method is specific, accurate, reproducible, and highly sensitive to be applied on the analysis of bulk form and capsules. Furthermore, the analysis requires a simple apparatus, thus the proposed method is suitable for routine analysis of LND in quality control laboratories.

## Abbreviations

LND: Lenalidomide; FLC: Fluorescamine; λ_ex_: Excitation wavelength; λ_em_: Emission wavelength; RFI: Relative fluorescence intensity; ICH: The international Conference on Harmonization; LOD: Limit of detection; LOQ: Limit of quantification; SD: Standard deviation; RSD: Relative standard deviation.

## Competing interests

The authors declare that they have no conflict of interests.

## Authors’ contributions

IAD proposed the subject, designed the study, participated in the results discussion and revised the manuscript. NYK participated in the assay design, conducted the validation of the assay, and participated in preparing the manuscript. AHB conducted the optimization of the assay conditions and prepared the draft version of the manuscript. NZA participated in study design, literature review, assay validation and preparing the manuscript. All authors read and approved the final manuscript.
